# A Macroscopic Interpretation of the Correlation between Electrical Percolation and Mechanical Properties of Poly-(Ethylene Vinyl Acetate)/Zn Composites

**DOI:** 10.3390/ma17112527

**Published:** 2024-05-24

**Authors:** Jerónimo Agrisuelas, Rafael Balart, José J. García-Jareño, Juan López-Martínez, Francisco Vicente

**Affiliations:** 1 Laboratory of Electrochemistry, Department of Physical Chemistry, University of Valencia, C/Dr. Moliner 50, E-46100 Burjassot, Spain; jeronimo.agrisuelas@uv.es (J.A.); jose.j.garcia@uv.es (J.J.G.-J.); 2 Instituto Universitario de Investigación de Tecnología de los Materiales (IUITM), Universitat Politècnica de València (UPV), E-03801 Alcoy, Spain; rbalart@mcm.upv.es (R.B.); jlopezm@mcm.upv.es (J.L.-M.)

**Keywords:** composite, ethylene vinyl acetate (EVA), zinc, physical properties, electrical percolation

## Abstract

Elastic composites were prepared using a procedure involving hot plates and zinc powder that was directly dispersed into an EVA matrix. The correlation between the zinc content and the conductive properties of the material was studied via impedance spectroscopy, the thermal properties of the material were studied via differential calorimetry and the mechanical properties of the composites were studied via tensile strength curves, representing an important advancement in the characterization of this type of composite material. The composites’ tensile strength and elongation at break decrease with the addition of filler since zinc particles act as stress-concentrating centres, while the composites’ hardness and Young’s modulus increase because of an increase in the stiffness of the material. The *AC* perturbation across the EVA/Zn composites was characterized using an RC parallel equivalent circuit that allowed us to easily measure their resistivity ($\rho_{p}$) and permittivity ($\varepsilon_{p}$). The dependence of these electrical magnitudes on the zinc content is correlated with their mechanical properties across the characteristic time constant $\tau_{p}$ = $\;\rho_{p}\cdot \varepsilon_{p}$ of this equivalent circuit. The dependence of the mechanical and electrical magnitudes on the zinc content is consistent with the formation of percolation clusters. The addition of graphite particles increases their potential performance. Three possible mechanisms for the electrical transport of the ac-perturbation across the EVA/Zn composites have been identified. Chemical corrosion in acid media causes the loss of zinc surface particles, but their bulk physical properties practically remain constant.

## 1. Introduction

Composite materials consisting of an isolator phase filled with a conductive powder are of special interest and have a wide range of particular uses [1,2,3,4,5,6,7,8,9,10,11,12,13,14,15]. When the filler content increases, the particles form inner structural clusters in the polymeric matrix. At one critical value of the filler volume fraction, an infinite framework conductive cluster is formed that crosses the whole of the material from one side to the other. This value is named “the percolation threshold” ($v_{pZn}$) [10,11] and depends on the components’ densities [16,17,18,19,20,21], filler size and shape, and the procedure used for preparing the samples of the composite material [22,23,24,25]. Over this critical value, their electrical ohmic character enables them to be used in sensors and electrochemical cells for the substitution of technological metals. Generally, the presence of nano-or micro-particles, which are susceptible to corrosion processes, necessitates studies of the effects of environmental corrosion phenomena on these composite materials to prevent limitations on their lifetime [26,27].

Zinc powder is a typical filler of composite materials with important technological applications and has been traditionally used to prevent corrosion in systems and in energy storage [28,29,30,31]. Poly-(ethylene-vinyl acetate) (EVA) is an elastic polymeric material with a low melting point, used intensively in the toy and footwear industries because of its combination of mechanical properties and easy processing. Thus, mixtures of both of these materials (EVA/Zn) are promising for various practical uses, including electromagnetic interference shielding [32], flame retardants [33], reinforced blends with halloysite nanotubes [34] and composite film heaters for effective de-icing applications [35], since it is well known that EVA composites do not cause significant levels of additional environmental damage [36]. They can be used in electrical applications and electronics due to their high electrical breakdown values, low dielectric loss, ease of processing and lower costs compared to other materials [37].

In this work, EVA was filled with increasing amounts of zinc powder, from 10 to 75 percent by weight. Before and after a chemical acid treatment, the samples were characterized by means of mechanical, calorimetric and electrical techniques. The aim of this work is to analyse the calorimetric and mechanical properties of EVA/Zn composites and correlate them with their electrical properties with the intention of expanding their use. For example, they could be utilized in new sensors, cell electrodes for energy storage, adhesives, anti-corrosion paints and so on, in addition to their technological uses in industrial sectors in which EVA or Zn are already used. The studied composites can be utilized in potential electronic applications as well as in anti-corrosion paints, automotive materials, sensors, capacitors and batteries, in particular. In recent years, EVA, as a part of blends or composites, has been increasingly used in construction applications due to its chemical-physical properties [38,39,40,41], as well as in diverse areas such as the flexible 3D printing fabrication of medical devices, allowing for a continuous, sustained and controlled local delivery of the incorporated drug with a single application [42,43]. Additionally, it is also used in filled biocomposites or in cenosphere composites [44,45]. Its increasing use in specific applications [46,47,48,49] accounts for research interest in its degradation by the effect of aggressive acidic media [50] as well as its use within strong dielectric fields [51], for de-icing applications [35] or as an agent of the compatibilization of polymer blends [52]. More generally, there is also interest in the evolution of its electrical and anticorrosive properties [53,54,55].

## 2. Materials and Methods

Polymeric matrix composites filled with metal powder were obtained by directly mixing EVA copolymer (20% vinyl acetate) (ALCUDIA PA-420 REPSOL, Puerto Llano, Spain) that was previously pulverized with 10, 20, 30, 40, 50, 60, 62.5, 65, 70, 72.5 and 75 percent by weight of zinc powder (MERCK, Madrid, Spain; L, particle size ˂ 63 µm) in plates that were 120 × 60 × 3.5 mm.

The mixtures were later put onto hot plates with a constant pressure of 10 kg·cm^−^^2^ for 180 s at 120°, followed with cooling to 40 °C for 600 s. The zinc mass fraction $m_{Zn}$ is related to zinc volume fraction $v_{Zn}$ as shown below:

(1) $$v_{Zn}=\frac{\frac{m_{Zn}}{\rho_{Zn}}}{\frac{m_{Zn}}{\rho_{Zn}}+\frac{\left(1-m_{Zn}\right)}{\rho_{EVA}}}$$

where $\rho_{Zn}$ and $\rho_{EVA}$ are the densities of Zn and EVA (7.14 and 0.91 g cm^−^^3^, respectively [56]).

AFM analysis was performed on a Multimode AFM microscope with a Nanoscope IIIa ADCS controller (Veeco Metrology Group, Cambridge, UK). A monolithic silicon cantilever (NanoWorld Pointprobe^®^ NCH) with a force constant of 42 N/m and a resonance frequency of 320 kHz was used in tapping mode.

Samples of 85 × 43 × 3.5 mm were cut and put into a glass container with 100 mL of 0.5 M KCl (Sigma Aldrch, Madrid, Spain) solution acidulated with HCl (PROLABO, VWR, Barcelona, Spain). The final pH was 4.5. The acid treatment consisted of five hours in acidulated water with acetic acid (pH = 4.5) at room temperature, stirring the samples in an ultrasound bath at 50 °C for 30 min every hour. After one hour, the solutions were adjusted to pH = 1.5 by addition of HCl. Afterwards, they were stirred for 30 min in an ultrasound bath at 50 °C.

To characterize their mechanical properties, the samples were removed from the original plates and from the corroded samples by the established procedure of UNE EN ISO 527-2:2012 [57]. Traction assays were performed using an IBERTEST ELIB 500 (Madrid, Spain) universal assay machine at 10 mm·min^−1^.

Thermogravimetric analysis of the EVA copolymers took place using METTER-TOLEDO TGA-SDTA 381e thermo-balance to determine the vinyl acetate content in the copolymers (20%). Experimental conditions were as follows: gas flux (20 mL·min^−1^), mass weight (10 mg) and the temperature ramp rate (from 30 to 700 °C at 10 °C·min^−1^).

To determine the transforming conditions, calorimetric studies of the samples in a METTER-TOLEDO DSC STAR-2000 calorimeter were carried out under the following conditions: gas flux (50 mL·min^−1^), mass weight (from 5 to 10 mg) and heat ramp (for 30 to 450 °C at 10 °C·min^−1^).

In order to avoid heating effects on the composite samples during EIS measurements, the applied potential and frequency were fixed. The electrical resistivity and permittivity were obtained by using an LCR Phillips PM 6304, selecting a frequency f=100 kHz and an amplitude of ∆E=±2V  with a superposed potential $E_{0}=2V$, because at these conditions, the ac-perturbation is strong enough but does not cause damage to the material over repeated measurements. Electrical contacts were made by using copper wires connected to two nickel electrodes of ***S*** = 9.0 cm^2^ placed in parallel to one another, symmetrically on each side of the composite. The distance between the two nickel electrodes is defined by the thickness of the composite samples *d* = 3.5 mm.

## 3. Results

### 3.1. DSC Characterization

**Figure 1:**
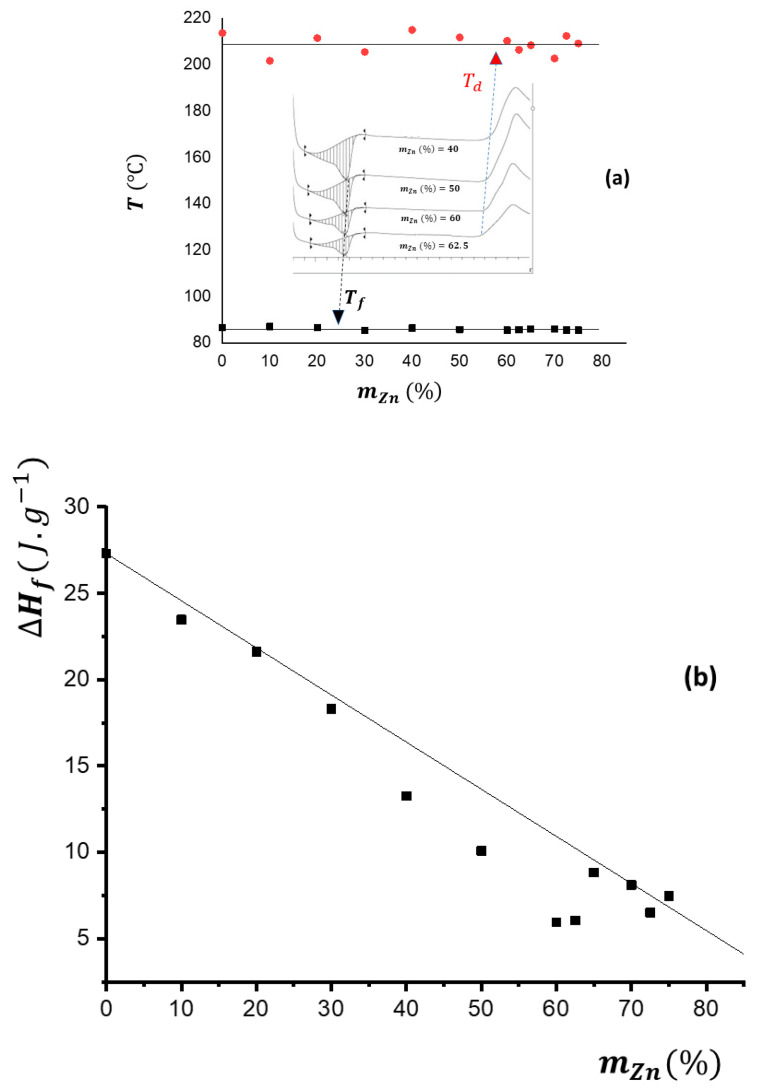
DSC characterization of the EVA/Zn composites with different contents of zinc: (**a**) Invariance in the temperatures of fusion (black squares) ***T_f_*** = 86 ± 1 °C and polymer degradation (red circles) ***T_d_*** = 210 ± 5 °C. Qualitative examples of DSC calorimetric curves where these characteristic temperatures are measured are shown in the central part. (**b**) Dependence of the fusion enthalpy Δ***H_f_*** (J/g) of the crystalline environments of EVA on the Zn content.

The melting temperatures $T_{f}\;$ of the crystalline environments of the EVA and the temperature at which the polymers began their thermal degradation $T_{d}\;$ remained constant at around 86 °C and 210 °C, respectively (Figure 1a), and were practically independent from the zinc content. Also, the enthalpy of fusion (per gram of composite) $∆H_{f}$ decreases approximately in a linear relationship as the proportion of zinc increases (Figure 1b), as is to be expected due to the dilution effect. However, there are small deviations from this linear relationship, in the range of 30 to 60 percent of the zinc content by weight (between $m_{Zn}\left(\%\right)=30\;$ and $m_{Zn}\left(\%\right)=60).$ This can probably be attributed to the orienting effect of the EVA chains by metal microparticles. This could be explained by the fact that the zinc is initially introduced into the amorphous zones of the EVA only, but once a certain concentration is reached, it begins to affect the crystalline zones. When zinc concentration values of about 60% by weight are reached, a saturated mixture is obtained.

### 3.2. Mechanical Properties

The filler acts inside the organic matrix (Table 1) in two ways, as can be observed from the dependence of the content of zinc on the mechanical properties. First, it acts as a reinforcement material on the shore hardness, reducing the mobility of the polymer chains by filler–polymer interactions, which cause the diminution of the elongation at break on the filler proportion, as is observed in others composites [58,59,60]. Additionally, zinc particles can potentially aggregate and act as stress-concentrating centres for the formation of cracks in the structure [61].

Nielsen proposed a model for the description of the decrease in elongation at break with the increase in the filler content in composite materials [62]. This model is based on spherical particles and assumes perfect adhesion between phases. In this case, the filler used for the composites’ preparation does not fulfil these conditions. In a homogeneous filler dispersion, the Young’s modulus increases with the proportion of filler due to restrictions imposed by rigid filler particles on the deformation of the polymeric matrix [63]. Several theoretical models have been proposed to describe the evolution of the Young’s modulus as a function of the filler content in composite materials. Two of the most important ones are the model proposed by Einstein that predicts a lineal relation between $\frac{F_{c}}{F_{p}}\;$ and the volume ratio v and the model of Guth [64] that predicts a parabolic relation, where $F_{c}$ and $F_{p}$ are the Young’s moduli of the composite material and the polymer matrix, respectively.

Also, the microparticles of zinc aggregate with interactions between metal particles being predominant over polymer–metal particle interactions, which cause a partial lack of adhesion between phases, as has been observed in other systems [26,41]. All these factors contribute to a great decrease in the elongation at break observed from the predicted values using Nielsen’s model.

**Figure 2:**
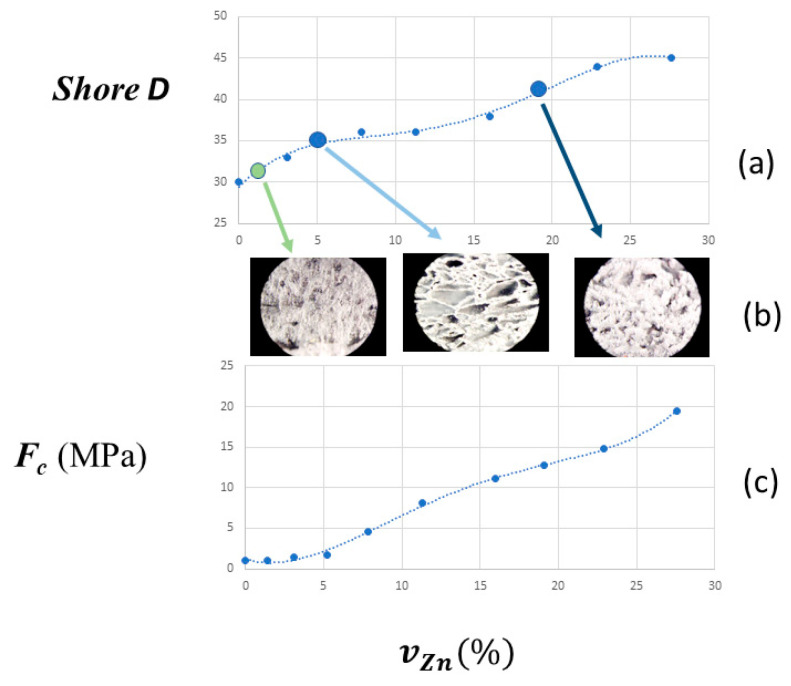
Dependence of some mechanical properties of the EVA/Zn composites on the zinc content. (**a**) Shore D dependence on $v_{Zn\;}\left(\%\right)$. (**b**) Optical images of the surface rupture of samples with 10, 30 and 62.5% Zn by mass (80% of magnification). (**c**) Dependence of the Young modulus $on\;v_{Zn}\left(\%\right)$. The same experimental conditions were used as those in Table 1.

Figure 2 shows the experimental dependences of the Young’s modulus of the composite vs. the filler concentration. We observed some deviations from the theoretical curves since Einstein’s model is valid for polymers filled with low amounts of non-interactive spherical particles, whereas Guth’s model takes into consideration the interactions between particles at higher filler concentrations, though it does not consider multiple particle interactions. In addition, in both cases, a homogeneous particle dispersion of the filler in the polymer matrix is supposed. None of these premises are fulfilled by the studied composites, but these models can be used to qualitatively describe the evolution of the Young’s modulus with an increasing filler proportion as part of a first approximation, as shown in Figures S2–S4 in Supplementary Materials.

**Figure 3:**
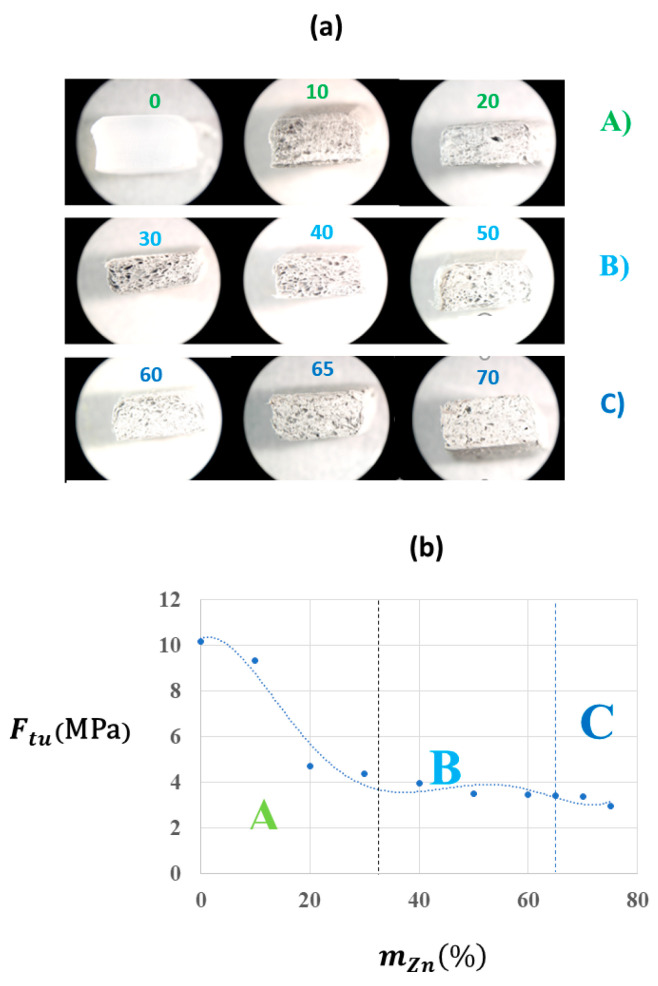
Detection of three ranges of surface rupture morphologies. (**a**) Optical images at 20% increments: within 0 to 30 (interval A), 40 to 60 (interval B) and greater than 60% zinc by mass (interval C). (**b**) Dependence of the ultimate tensile strength ***F_tu_*** on the zinc content in percentage by weight. The same conditions as those in Table 1 were used.

In the case of the EVA/Zn composites, their Young’s modulus and surface hardness increase with the zinc content (Figure 2), as predicted by both theoretical models. However, in the intermediate range of $m_{Zn}\left(\%\right)=$30 to $m_{Zn}\left(\%\right)=$60 or between $v_{Zn}\left(\%\right)=5.2$ and $v_{Zn}\left(\%\right)=16$, respectively, the surface Shore D hardness values are lower than expected according to the general trend over the entire range, and the opposite is observed for the values of the Young’s modulus. This suggests that the zinc particles are statistically distributed but form structures that simultaneously contribute to the increase in the surface elasticity and the internal stiffness of the composite samples. This hypothesis is consistent with the observed morphology of the rupture surfaces, because, in this region of intermediate compositions, the optical images show defined cells with a spongy appearance, compared to the morphology of the rupture surfaces of the lower or higher contents (Figure 3a). All of our calorimetric results and optical microscopic observations are consistent with the existence of three ranges of the zinc content in the double logarithmic plot of the Young’s modulus in front of the volume ratio (Figure 3b). The addition of the metallic filler diminishes the elasticity of the composite. Zinc particles were dispersed in the polymeric matrix and aggregates were formed that act as stress-concentrating centres, favouring the rapid propagation of cracks during stress and limiting the elastic deformation of the matrix.

### 3.3. Electrical AC Characterization

From a phenomenological perspective, the percolation threshold corresponds to the content of zinc at which the electrical regime changes from conduction by polarization through the dielectric polymeric phase to the conduction of electrons through the network of metal particles that are in contact.

**Figure 4:**
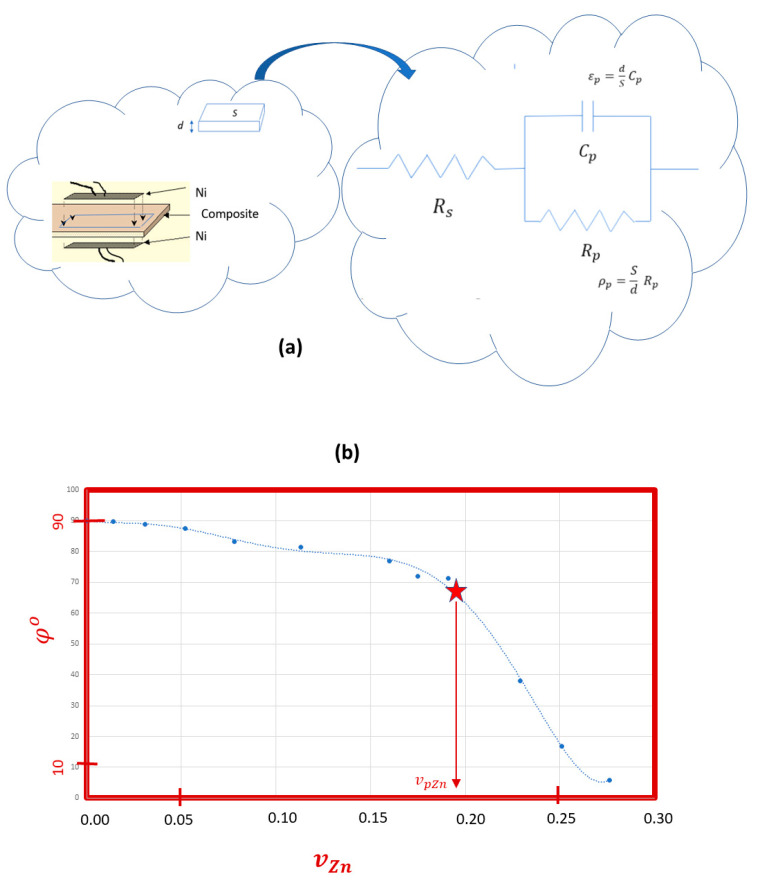

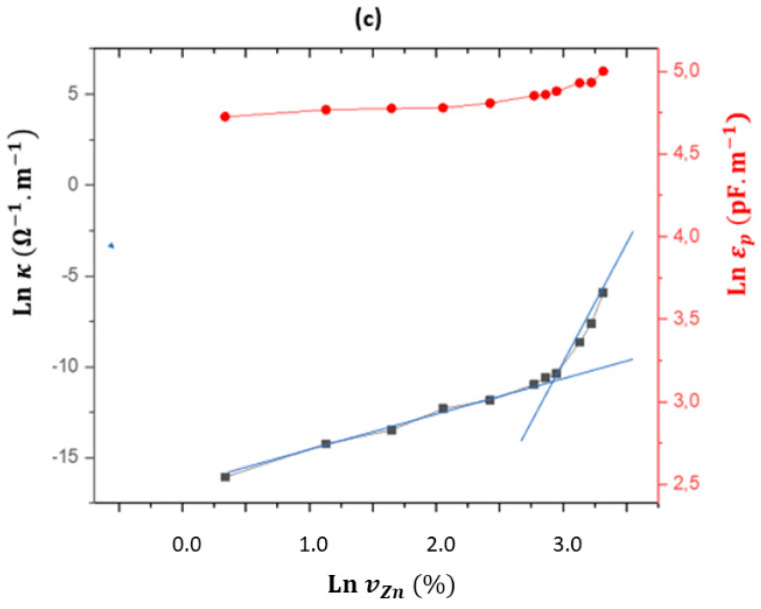
Transition from a polarization regimen to a framework cluster of conducting particles of the ac-electrical conduction with the increase in the zinc content. (**a**) Diagram of the measurement cell and diagram of the equivalent RC in parallel circuit to measure the resistivity $\rho_{p}$ and permittivity $\varepsilon_{p}$. (**b**) Dependence of the phase angle $\varphi^{o}vs.v_{Zn}$. The red star highlights the drastic change of the phase angle at the percolation threshold. (**c**) Comparative dependences of permittivity (red circles and red axis) and conductivity (black squares) on the zinc content. The straight lines on the conductivity curve only indicate trends. The same conditions as those in Table 2 were used.

By applying a sinusoidal potential perturbation in a sandwich cell to the material, the following is obtained (Figure 4a):

(2) $$E=E_{0}+∆Esen(2\pi ft)$$

with a large enough surface S for the two electrodes, which are separated at a distance d corresponding to the thickness of the composite sample, an model of RC equivalent circuit is very practical for calculating the resistivity $\rho_{p}$ as follows:

(3) $$\rho_{p}=R_{p}\frac{S}{d}$$

and the permittivity $\varepsilon_{p}$ is as follows:

(4) $$\varepsilon_{p}=C_{p}\frac{d}{S}$$

where the resistance $R_{p}\;$ and capacitance $C_{p}$ are measured directly using the RCL instrument.

Even though EIS is a powerful technique for characterizing materials. From a practical perspective, measuring their electrical magnitudes at a constant frequency could be enough as a first approximation of the development of these composite materials as well as for control of their fabrication (Table 2).

Selecting a frequency of f=100 kHz , an amplitude of ∆E=±2 V and a superposed potential of $E_{0}=2V$, the phase angle $\varphi^{o}$ of the current intensity response decreases drastically from the zinc content corresponding to the percolation threshold $v_{pZn}=0.19\;$ (Figure 4b). At this critical value, the law of dependence of the conductivity $\kappa =\frac{1}{\rho_{p}}$ on the ratio of zinc also changes, since it is the intercept of the two linear fits of Ln κ vs. Ln $v_{Zn}$ (Figure 4c), which corresponds to a change in the *ac* regime of the flow through the samples from ac-polarization to dc-resistance. At $v_{Zn}\leq v_{pZn}$, ***κ*** $\propto \left(v_{p}\right)$^2.2^, whereas ***κ*** $\propto {\left(v_{Zn}-v_{pZn}\right)}^{t\gg 3}$ at $v_{Zn}\geq v_{pZn}$.

The composites act as dielectric Insulating thermoplastics at low zinc contents and ohmic conductors at high zinc contents above their percolation threshold.

From a fractal geometry perspective, the system can be idealized as a distribution of an elevated number of plane-parallel plate micro-capacitors inside a 3D space, in which the dielectric is formed by the non-conducting polymer matrix and the conducting microplates are formed by the metallic zinc particles. It can be imagined that the total system capacitance and dielectric permittivity increase as the total surface area of the microplates increases, while the microscopic distance between the plates decreases.

**Figure 5:**
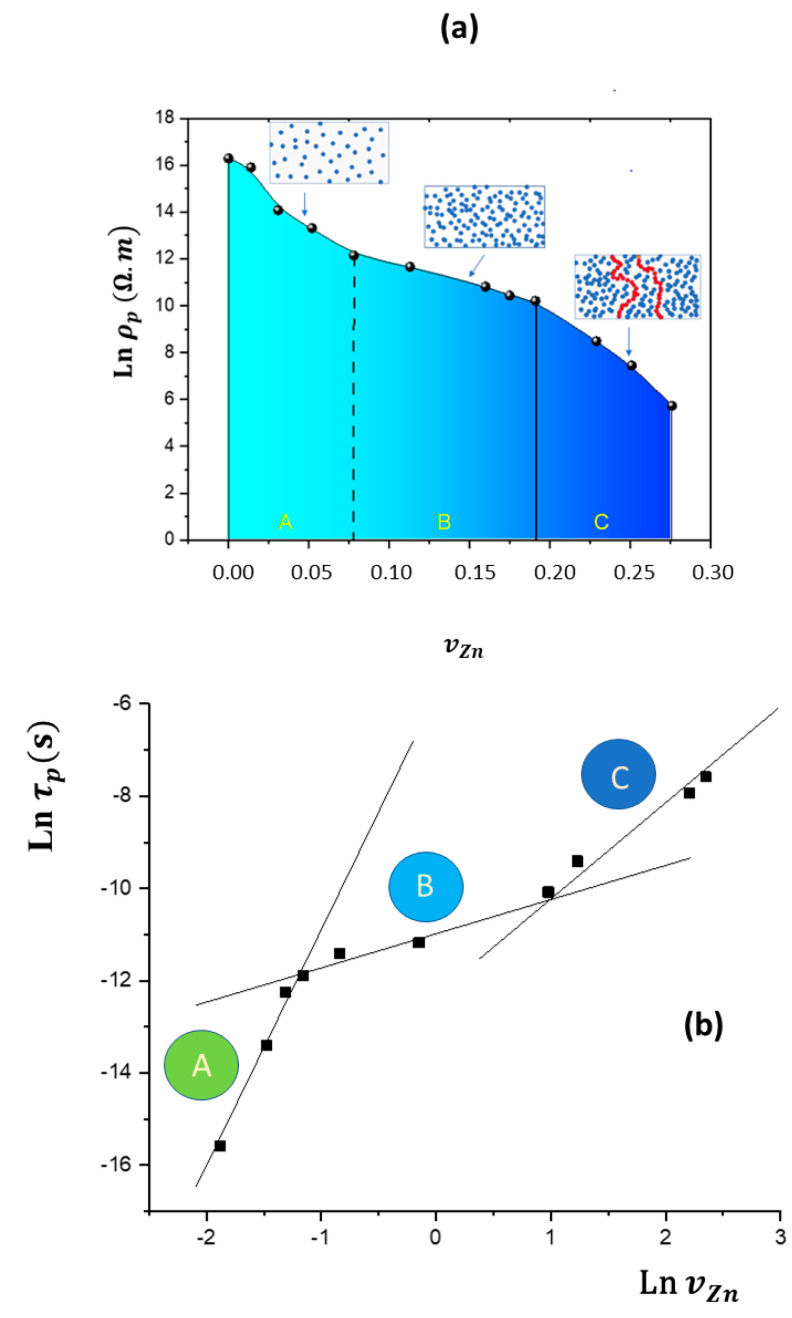
Observation of three resistivity and permittivity ranges of the composite according to the zinc content. (**a**) Dependence of $\rho_{p}$ on $v_{Zn}$. Insets are idealistic representations of 5, 15 and 25% spherical metallic particle volumes. (**b**) Dependence of $\tau_{p}$ on $v_{Zn}$. The straight lines above the experimental points in zones A, B and C show the three respective conductivity trends. The same conditions were used as those in Table 2.

The logarithm of the resistivity decreases with the volume fraction of zinc particles, showing three clearly differentiated zones depending on the propagation regime of the AC perturbation (Figure 5). A singular point is observed in the curve of this figure around $v_{Zn}=0.075$, which may be related to the interaction between the zinc particles and the EVA matrix. Then, an intermediate interval between $v_{Zn}=0.075$ and $v_{pZn}=0.19\;$ is phenomenologically observed that coincides with the intermediate interval of the values of some of the mechanical magnitudes (Figure 2): the experimental tensile strength and the elongation at break are greatly decreased when the zinc content is increased (Table 1).

These observations lead us to postulate that the self-association of the zinc particles increases the area of the micro-capacitor plates but also decreases the distance between neighbouring micro-capacitors. This suggests that, as the proximity between them increases, the series association will have a greater impact on the total permittivity value. Therefore, the change in the polarization regime postulated for $v_{Zn}=0.075\;$ must correspond to the phenomenon of approximation between the conductive particles and consequently to the dependence of the parallel/series distribution of the micro-capacitors on the content of zinc microparticles.

The aforementioned mechanical and electrical results show the dispersion of the zinc filler was not completely aleatory in the polymer binder, and aggregates were formed at low zinc volumes. Perhaps the acetate groups prefer to envelop zinc particles, orientating the percolation cluster formation and growth, as has been observed in other similar composite materials [65].

For the non-homogeneous complete dispersion of the filler in the polymer matrix, interactions between filler particles play a more important role in the mechanical properties of this material. In these cases, the increase in Young’s modulus is mainly caused by the contact of the particles with one another. This is because the moduli of the zinc are bigger than those of the polymer matrix. Aggregates act as a continuous fibrous filler that increases the stiffness of the material, as reflected in the Young’s modulus, even though the inclusion of metallic particles produces an increase in the hardness of the material [66]. In Figure 4 and Figure 5, it is observed that over a zinc content volume $\;v_{pZn}=0.19$, the behaviour of the composite is clearly resistive, corresponding to that of a *dc* conducting material. This proportion, a volume that is greater than 60% by weight, can be inconvenient for some applications of the composite material due to its high density. One solution is to manufacture ternary composites with carbon fillers to decrease their density or increase their electrical conductance [67,68,69], e.g., in graphite composites (40% by weight EVA/50% graphite/10% Zn) (last row of Table 2). This is only one example of the enormous number of possibilities for modifying this EVA/Zn composite for specific uses. However, the oxidation behaviour of its surface particles is similar to that of zinc metal [70,71], and its physical properties qualitatively approach those theoretically predicted by means of biphasic composite models [72].

The measurement of the dielectric permittivity and resistivity of composite samples is highly dependent on the experimental conditions and on the electrical perturbation applied. However, by trying to use accurate experimental conditions, the effect of the content of conductive particles in an insulating polymer matrix can be quantified. Particularly interesting is the measurement of $\tau_{p}$ = $\rho_{p}\cdot \varepsilon_{p}$, which intuitively indicates the speed at which the alternating current electrical perturbation percolates through the samples.

## 4. Discussion

By replacing some zinc particles with carbonaceous particles, such as graphite, it is possible to design materials with a wide range of physical characteristics for use in specific applications. A low metal content is sufficient for such ternary composite materials to exhibit a sufficient level of conductivity to be used as electrodes in electrochemical cells [72,73,74]. By replacing the metal itself with carbonaceous particles, including the use of pyrolyzed vegetable fibres [75], foams [76] or different carbon particles [77,78,79,80], it is possible to enable the design of materials at the level of performance required for their specific technological applications. In this sense, it is noticeable that the dispersion of zinc microparticles in increasing proportions in the EVA polymeric matrix increases the electrical conductivity of the composite, although it was observed that only the metallic microparticles in contact with the medium for all the proportions tested appear to suffer from chemical corrosion, since the electrical propagation processes for the material were not altered because the polymeric matrix acts as a protective barrier against the corrosion of the particles inside.

**Figure 6:**
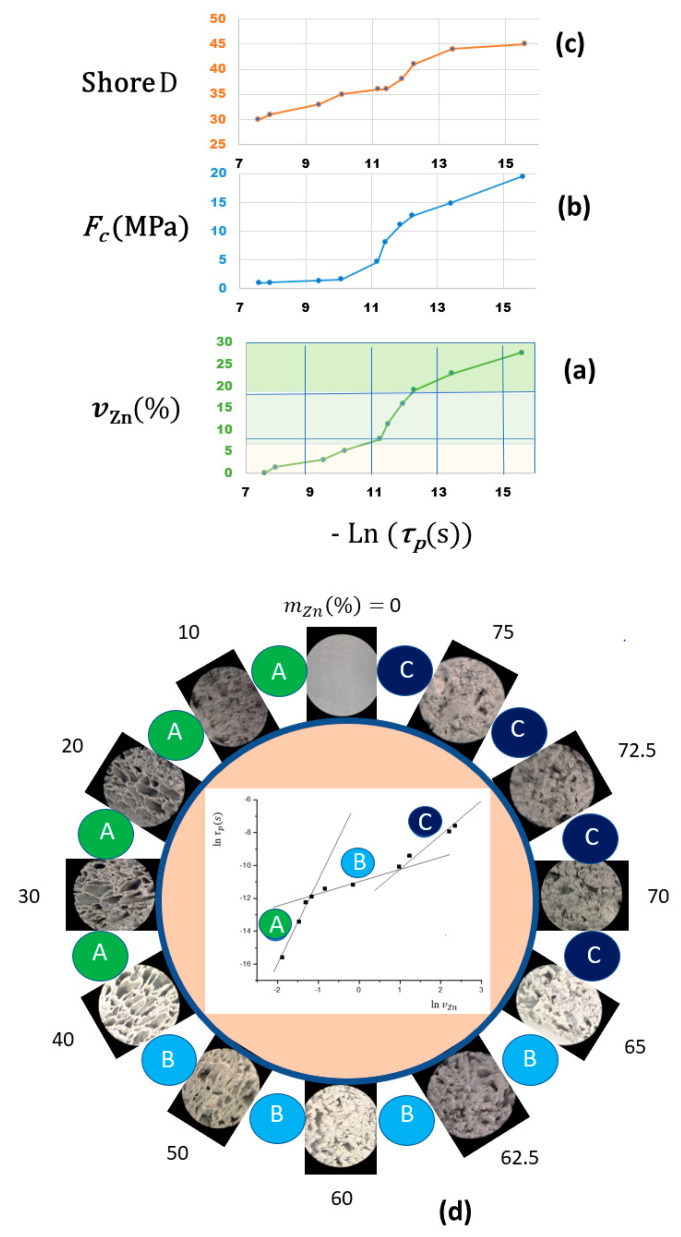
Some correlations between the dependences of electrical and mechanical properties on the zinc content. Dependence of $Ln(\tau_{p})$ on (**a**) $v_{Zn}$, (**b**) on $F_{c}$ and (**c**) on Shore D. (**d**) Optical images of the surface rupture of the three ranges of electrical and mechanical properties (80× magnification). The same conditions were used as those in Table 1 and Table 2.

The composites act as dielectric Insulating thermoplastics at low zinc contents and ohmic conductors at high zinc contents above the percolation threshold. The dependence of the resistivity of the samples on the zinc microparticle content shows three intervals (Figure 5b), which must be related to the formation of electrical percolation clusters. For low contents, the conduction is accounted for by the polarization mechanism, and for contents above 62.5% in weight ($v_{Zn}\left(\%\right)\geq 19),$ the behaviour is clearly resistive, so it is assumed that this content can be considered as the threshold for effective percolation from a phenomenological perspective. In the intermediate content region, it can be postulated that in addition to polarization conduction, a tunnelling current flow occurs, assuming that the zinc microparticles are randomly dispersed in the polymer matrix with low contents of metal. However, there are some additional effects, as the enthalpy of the fusion data deviates from linearity, which is expected due to the dilution effect (Figure 1b). Also, the Young’s modulus shows lower values for parabolic dependence, which correspond to Guth’s model. All these data point to the fact that within this two-phase composite, there is a tendency towards microstructures formed by zinc particles, due to a greater interaction between the particles than with the polymeric matrix. From what we can deduce from the optical images of the material’s rupture surface, the cells decrease in number and size as the proportion of zinc increases. This suggests that, in addition to the tunnelling effect, a structural effect is produced as the percolative paths of the electric current are formed by increasing the proportion of zinc particles. Hence, there is an analogous correspondence between the dependencies of the mechanical magnitudes and the electrical time $\tau_{p}$ on the content of zinc (Figure 6).

It has been detected by electrical measurements that there are three possible mechanisms for the electrical transport of the *ac* perturbation across the EVA/Zn composites: (A) conduction by polarization of the dielectric at low zinc concentrations; (B) conduction by the tunnelling effect together with polarization at intermediate contents; and (C) ohmic dc conduction through the network of conductive particles that are in contact above the effective percolation threshold (Figure 6d). These ranges are directly evident in the optical images of the breakdown surface (Figure 3 and Figure 4) since the surfaces show less homogeneity in the intermediate interval.

At a lower level of filler loading, $v_{Zn}\leq 0.075$, the filler particles remain isolated within the matrix. Then, the polarization of the polymer is increased by the polarization of the interface of the Zn particles/EVA polymers due to the electrical linear field generated between the two parallel nickel electrodes. Therefore, the increase in the permittivity with the zinc content with respect to the EVA could be related to the parallel association of the micro-capacitors dispersed into the dielectric EVA medium with the capacitor of the sandwich cell.

At an intermediate level of filler loading, $0.075\leq v_{Zn}\leq 0.19$, the filler particles form a three-dimensional network of micro-capacitors with a wide distribution of geometrical shapes and sizes, in which the number of interactions between them increases as the number of zinc particles increases. It seems that their self-association evolves in series to a formation with percolative dc-conducting clusters through the increase in the association of the micro-capacitors.

At a high level of filler loading, above the percolation threshold of v≥0.19, the current flows preferably across the metal particles that are in contact because of the formation of continuous conductive networks. Even though there are consistencies between the EVA/Zn composite’s calorimetric, optical, mechanical and electrical properties, a rigorous correspondence between its phenomenological behaviour and theoretical interpretations has not yet been reached. However, theoretical concepts of percolation are very useful for explaining the aforementioned results from a practical, comparative perspective.

**Figure 7:**
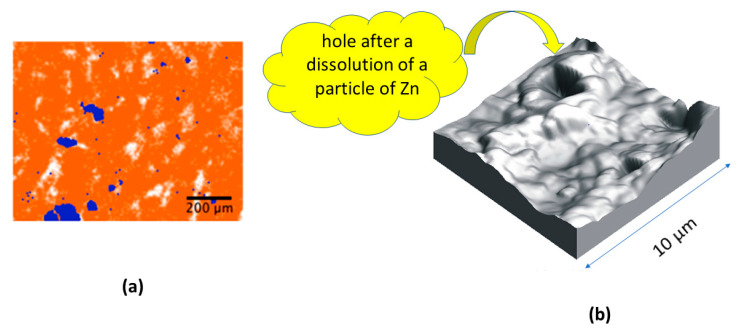
(**a**) Aleatory distribution of Zn particles (in blue) from an EDX microanalysis of the surface of a composite with a 10% content by weight. (**b**) 3D plot of AFM of EVA sample with 20% Zn by weight with an image size of 10 × 10 µm after the acid treatment.

The surface of the composite samples shows microparticles of zinc (Figure 7a). The treatment of the samples in an acidic aqueous medium removes these surface particles of zinc, leaving instead voids that contribute to an increase in the roughness of the samples’ surfaces. Acidic media attacks cause an increase in the number of valleys and peaks with a consequent increase in surface roughness (Figure 7b). In the top right image obtained by AFM, the dissolution of a zinc microparticle after an acid attack is clearly observed. This fact is consistent with the decrease in the surface zinc content showed by an EDX elemental microanalysis and optical images of EVA/Zn samples with different zinc contents [81]. In acidic media, the surface microparticles of zinc are dissolved, thus increasing the surface roughness of the composite. This phenomenon is a well-known heterogeneous redox process in the corrosion of metallic zinc, but in this case, the polymeric matrix of the composite protects the microparticles inside it from the chemical attacks of the environmental medium. It was also observed that the surface corrosion of all samples caused by the acid treatment did not noticeably affect their physical properties, since there are practically no differences between the treated and untreated samples (see Figures S1–S4 and Table S1 in Supplementary Materials). Further research on the corrosion kinetics and durability of these types of materials requires the use of coupled techniques [82] to simultaneously obtain their electromagnetic, mechanical and thermodynamic properties in order to improve their use in technological applications. The oxidation and dissolution of the zinc particles on the surface of the EVA/Zn composite caused by the acidic aqueous environment can be considered part of the accelerated corrosion tests that simulate many environmental media. This corrosion causes deterioration by the loss of surface zinc that does not affect the interior of the composite. The EVA matrix maintains its chemical and thermal stability at the tested ratios. It is corroborated that the heterogeneous corrosion on the surface does not progress across the composite samples since their calorimetric, mechanical and electrical properties remain practically constant after the acid treatment. Since the zinc corrosion occurs on the outer faces of the composite samples, the rest of the particles remain inside the polymer matrix and can also act as sacrificial anodes if the matrix suffers environmental damage.

## 5. Conclusions

The dispersion of zinc powders in a polymeric matrix is a model of an insulating polymer/conducting load composite in which its mechanical properties correlate with its electrical properties.

It has been detected by electrical measurements that there are three possible mechanisms for the electrical transport of the *ac* perturbation across EVA/Zn composites: (A) conduction by polarization of the dielectric at low zinc concentrations; (B) conduction by the tunnelling effect together with polarization at intermediate zinc contents; and (C) ohmic dc conduction through the network of conductive particles that are in contact above the effective percolation threshold.

The EVA/Zn composite material shows promising mechanical and electrical properties for some new technological and practical uses, which can be improved if part of the zinc content is changed by carbon particles, reducing the composite’s density and controlling its electrical properties for specific uses.

## Figures and Tables

**Table 1:** Dependence of some mechanical magnitudes on the content of zinc: shore hardness, unitary elongation at break $\left(\frac{∆l}{l_{o}}\right),$ ultimate tensile strength ($F_{tu}$) and Young’s modulus (*F_C_*). Ibertest ELIB 500. v = 10 mm·min^−1^.

$m_{Zn}$	$v_{Zn}$	Shore D	$\frac{∆l}{l_{o}}$	$F_{tu}$	*F_C_*
(%)	(%)		(%)	MPa	MPa
0	0	30	10.47	10.18	0.97
10	1.4	31	9.11	9.34	1.03
20	3.1	33	3.43	4.73	1.38
30	5.2	35	2.66	4.36	1.64
40	7.8	36	0.86	3.97	4.62
50	11.3	36	0.43	3.49	8.08
60	16.0	38	0.31	3.48	11.09
65	19.1	41	0.27	3.44	12.71
70	22.9	44	0.23	3.40	14.86
75	27.6	45	0.15	2.98	19.51

**Table 2:** Measured electrical magnitudes of EVA/Zn composites with different amounts of Zn particles. **T** = 298 K, Eo=2V, ∆E=±2V, and f=100 kHz. The last correspond to two samples with graphite powder contents of $m_{C}$.

$m_{Zn}$ (%)	$m_{C}$ (%)	$\varphi^{o}$ Degree	$\rho_{p}$ Ω·m	$\varepsilon_{p}$ pF·m^-1^	$\tau_{p}$ µs
0	0	89.9	1.19 × 10^7^	43	510
10	0	89.8	8.13 × 10^6^	44	360
20	0	88.9	1.30 × 10^6^	63	82
30	0	87.4	6.09 × 10^5^	69	42
40	0	83.2	1.89 × 10^5^	70	14
50	0	81.5	1.17 × 10^5^	92	11
60	0	77.0	4.99 × 10^4^	137	6.9
62.5	0	72.0	3.39 × 10^4^	145	4.9
65	0	71.2	2.73 × 10^4^	175	4.8
70	0	38.1	4.87 × 10^3^	300	0.15
75	0	5.8	3.09 × 10^2^	544	0.17
0	50	72.0	1.83 × 10^4^	267	4.9
10	50	20.6	5.74 × 10^2^	1000	0.57

## Data Availability

The original contributions presented in the study are included in the article, further inquiries can be directed to the corresponding author.

## Supplementary Materials

The following supporting information can be downloaded at https://www.mdpi.com/article/10.3390/ma17112527/s1, Figure S1. Optical and AFM images, before and after acid treatment. Figure S2. Unitary elongation at break for all composite samples before and after acid treatment. Figure S3. Ultimate tensile strength ($F_{tu}$) for composite samples, before and after acid treatment. Figure S4. Experimental relative Young’s modulus for composite samples before and after acid treatment. Table S1. Shore Hardness for all composite samples before and after acid treatment.
