# Supplementary material for: A Macroscopic Interpretation of the Correlation between Electrical Percolation and Mechanical Properties of Poly-(Ethylene Vinyl Acetate)/Zn Composites
Source: Materials (Basel). 2024 May 24;17(11):2527. doi: 10.3390/ma17112527 (PMC11173855; doi:10.3390/ma17112527)
Supplement: Supplementary file 1 [file materials-17-02527-s001.zip › materials-2964395-supplementary.pdf]

Supplementary Materials

A macroscopic interpretation of the correlation between electrical percolation and mechanical
properties of poly- (ethylene vinyl acetate)/Zn composites

Jerónimo Agrisuelas [1], Rafael Balart [2], José J. García-Jareño[1], Juan López-Martínez [2], Francisco Vicente[1*]

[1] [1]Laboratory of Electrochemistry. Department of Physical Chemistry, University of Valencia. C/ Dr. Moliner 50, E-46100 Burjassot, Valencia (Spain).
[2] Instituto Universitario de Investigación de Tecnología de los Materiales (IUITM), Universitat Politècnica de València (UPV), E-03801 Alcoy, Spain
* Correspondence: francisco.vicente@uv.es

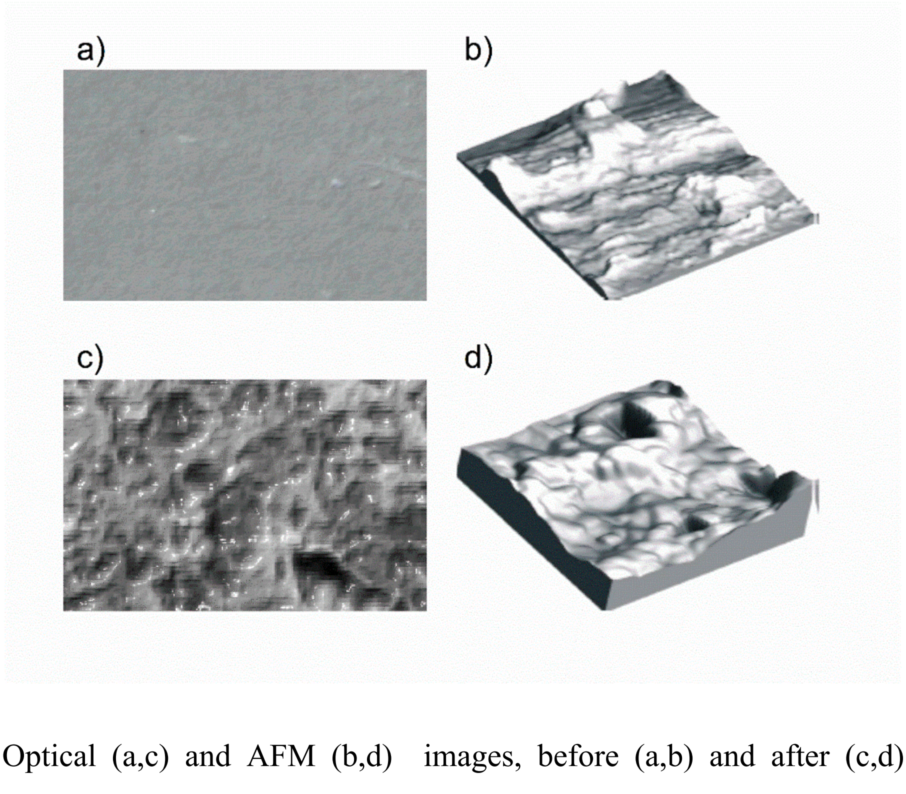

Figure S1. Optical (a,c) and AFM (b,d) images, before (a,b) and after (c,d) acid treatment**.** RMS roughness values for EVA samples $m_{Zn}(\%) = 20$ without treatment and with acid treatment for two different sizes (AFM).

	Sample 10 x 10 µm			Sample 30 x 30 µm		
	Rms	Average height (Å)	Max Height (Å)	*Rms*	Average height (Å)	Max Height (Å)
Untreated	193	445	1158	342	662	2397
Treated	410	1664	3485	702	2008	4381

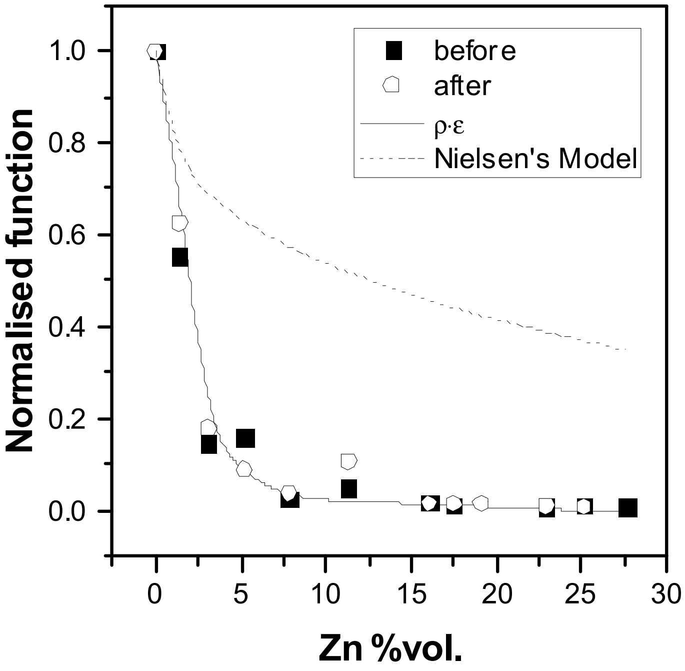

Figure S2. Unitary elongation at break for all composite samples before (■) and after (○) acid treatment. Comparison with the predicted curve calculated with Nielsen's model (solid line) and with the relative $\tau_{p=}\rho_p.\, \varepsilon_p$ (dashed line). Ibertest ELIB 500. v=10 mm·min^{-1}.

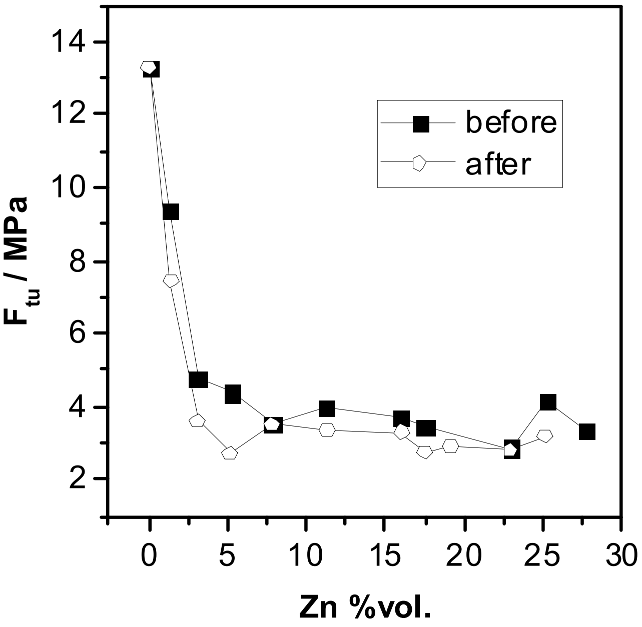

Figure S3. Ultimate tensile strength (F_{tu}) for composite samples, before (■) and after (○) acid treatment performed by an Ibertest ELIB 500 at 10 mm·min^{-1}.

Table S1. Shore Hardness for all composite samples before and after acid treatment

v_{Zn} (%)	**Shore D** Without treatment	**Shore D** After acid treatment
0	30	30
1.4	31	31
3.1	33	32
5.2	35	33
7.8	36	34
11.3	36	36
16.0	38	40
17.5	40	39
19.1	41	41
22.9	44	45
25.1	43	45
27.6	45	46

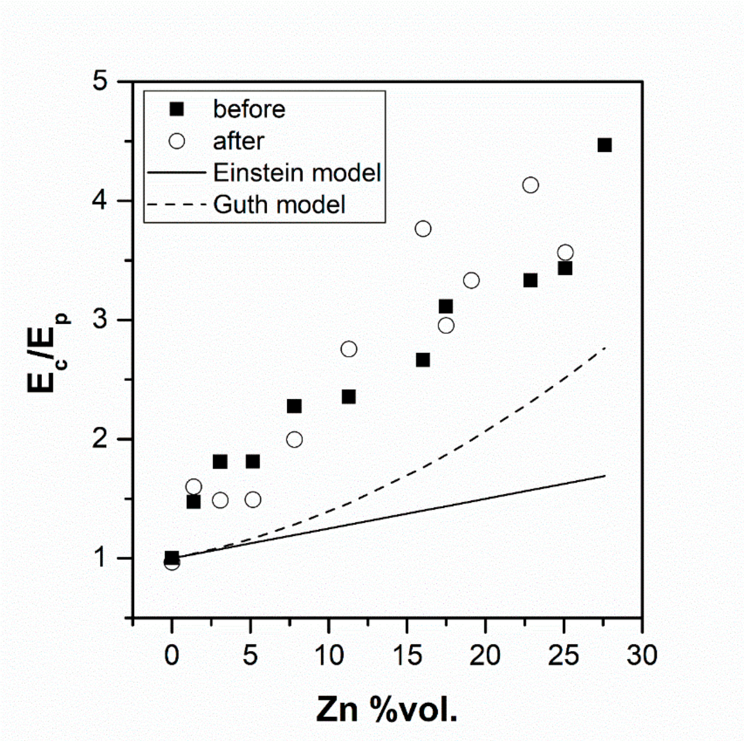

Figure S4. Experimental relative Young's modulus for all composite samples before (■) and after (○) acid treatment. The solid line is the Einstein model and the dashed lines is the Guth model . Ibertest ELIB 500. v=10 mm·min^{-1}. F_p=30MPa.
